# National Trends and Impact of the COVID-19 Pandemic on Trauma Patient Care in the United States

**DOI:** 10.7759/cureus.97672

**Published:** 2025-11-24

**Authors:** Nzi Jacques Philippe Niamien, Oluwayemisi Oyeleke, Chin-I Cheng, Akram Alashari

**Affiliations:** 1 General Surgery, Central Michigan University College of Medicine, Saginaw, USA; 2 Statistics, Actuarial and Data Science, Central Michigan University College of Medicine, Saginaw, USA

**Keywords:** covid-19, healthcare access disparities, healthcare disparities, retrospective studies, trauma severity indices, wounds and injuries

## Abstract

Introduction

The COVID-19 pandemic disrupted healthcare delivery worldwide. We examined whether national trauma demographics, injury patterns, severity, comorbidities, in-hospital events, procedures, and outcomes differed before and during the pandemic using a large multicenter dataset in the United States.

Methods

We performed a retrospective analysis of the American College of Surgeons Trauma Quality Improvement Program dataset. The pre-pandemic years were 2018-2019, and the pandemic year was 2020. Variables included patient demographics, mechanism and type of injury, prehospital and early in-hospital care, comorbidities, in-hospital events, procedures, length of stay, and mortality. Continuous variables were summarized as medians with interquartile ranges, and categorical variables were summarized as frequencies with percentages. Associations across years were tested using chi-square tests for categorical variables and one-way analysis of variance for continuous variables. Statistical significance was set at p≤0.05.

Results

Year-to-year differences were observed across multiple domains. The demographic distribution shifted, with a higher proportion of Black patients and lower proportions of White and Asian patients during the pandemic year. Penetrating trauma increased, while blunt trauma decreased slightly; mechanisms such as cutting/piercing and gunshot wounds became more common, whereas motor-vehicle occupant injuries declined. Indicators of acuity suggested sicker presentations, including more early blood transfusions and a smaller share of low injury severity score (ISS) cases. Comorbidities and patient characteristics also shifted, with higher proportions of alcohol use disorder, mental health disorders, smoking history, and functional dependence. In-hospital events showed increases in cardiac arrest, pulmonary embolism, unplanned intubations, and unplanned intensive care unit admissions, while rates of deep surgical site infection, deep vein thrombosis, compartment syndrome, organ-space infection, osteomyelitis, and stroke were stable. Operative rates for thoracotomy and laparotomy and lengths of stay were unchanged, whereas mortality was higher during the pandemic year.

Conclusions

This national analysis shows that the pandemic materially altered trauma epidemiology, case mix, and in-hospital course, with greater penetration of violent mechanisms, higher acuity at presentation, and worse outcomes. These findings highlight vulnerability points in trauma systems and support actionable priorities for practice: strengthening surge preparedness and critical-care capacity; ensuring reliable blood product availability; integrating violence-prevention, mental health, and substance-use screening into trauma pathways; and advancing equity-focused policies to safeguard access and outcomes for underserved populations.

## Introduction

The COVID-19 pandemic has disrupted healthcare systems worldwide since early 2020. According to the WHO, more than 770 million cases have been reported to date, resulting in millions of hospital admissions and a death toll of 7.1 million [1]. In the US, the pandemic prompted substantial policy and hospital-management changes. Multiple health systems have reported the pandemic’s effects on trauma admissions and outcomes using institutional data. Although the impact varied by geography and level of care, several patterns emerged. This study aimed to identify national trends using the Trauma Quality Improvement Program (TQIP) database, which aggregates data from multiple trauma centers across the US.

## Materials and methods

We conducted a retrospective analysis of the TQIP dataset. Data from 2018, 2019, 2020, and 2021 were available at study initiation. We categorized 2018-2019 as pre-COVID years and 2020 as the COVID-19 pandemic year; primary comparisons focused on these three years (2018-2020). Institutional Review Board approval was not required because the dataset contained only de-identified patient information. We evaluated differences in patient demographics, mechanism of injury, injury type, prehospital information, in-hospital procedures, pre-existing conditions, in-hospital events, and patient outcomes before and during the pandemic. The data obtained from the database were analyzed in full without excluding any data points. We intentionally omitted 2021 data to focus on early pandemic effects on the health system. Future studies should examine later pandemic outcomes and the impact of new treatment modalities and vaccination on disease management.

Statistical analyses were performed using SAS 9.4 (SAS Institute Inc., Cary, NC, USA). We summarized continuous variables as mean (standard deviation) and categorical variables as frequency (percentage). Chi-square tests were used to assess the associations between categorical variables and the year. One-way analysis of variance tested differences in means across years for average age. Age was summarized as the mean with standard deviation. A Kruskal-Wallis test assessed differences in length of stay (LOS) across the three years due to skewed LOS data. Length of stay was summarized as medians with interquartile ranges. Because the skewness values for 2018, 2019, and 2020 all fell within −0.2 to 0, and the kurtosis values were within −1.2 to 0, the data appeared approximately symmetric and near normal. We considered results statistically significant at P ≤ 0.05.

The American College of Surgeons (ACS) maintains the TQIP dataset to promote standardized trauma care nationwide. Participating trauma centers submit data based on the National Trauma Data Standard (NTDS), established in 2007 by the ACS Committee on Trauma, to ensure uniform data elements. The NTDS enables hospitals to benchmark outcomes and supports research to identify national trends [2]. Trauma centers participating in TQIP submit data regardless of their trauma center level.

## Results

Demographics

The proportion of male patients differed significantly across years: 59.25% (618,310/2018), 58.91% (646,259/2019), and 59.95% (680,322/2020). Age distribution differed significantly. Race/ethnicity proportions differed significantly. Black patients comprised 15.58% (173,464/2020) of the cohort, compared with 14.30% (154,277/2019) and 14.20% (145,790/2018). In 2020, White and Asian patients comprised 73.93% (823,139) and 1.78% (19,872), respectively, both lower than in prior years (Table 1).

**Table 1:** Demographics, injury type, and mechanism of injury by year The p-values were calculated using chi-square tests for categorical variables and one-way ANOVA for continuous variables. The p-value shown pertains to the overall comparison across subcategories. MVA: Motor vehicle accident

Variable		Year			p-value
		2018	2019	2020	
Mean age, years (standard deviation)		49 (24.6)	49.5 (24.7)	48.8 (24.6)	<0.0001
Age group	≥18 years, n (%)	864,159 (82.79%)	906,693 (82.64%)	938,321 (82.67%)	0.006
	<18 years, n (%)	179,577 (17.21%)	190,497 (17.36%)	196,697 (17.33%)	
Sex	Male, n (%)	618,310 (59.25%)	646,259 (58.91%)	680,322 (59.95%)	<0.0001
Race/ethnicity	White, n (%)	771,325 (75.13%)	810,045 (75.08%)	823,139 (73.93%)	<0.0001
	Black, n (%)	145,790 (14.20%)	154,277 (14.30%)	173,464 (15.58%)	
	Asian, n (%)	19,808 (1.93%)	21,106 (1.96%)	19,872 (1.78%)	
	Other, n (%)	89,797 (8.75%)	93,550 (8.67%)	96,970 (8.71%)	
Injury type	Blunt, n (%)	902,488 (88.06%)	960,230 (88.39%)	973,047 (86.60%)	<0.0001
	Penetrating, n (%)	91,365 (8.92%)	95,484 (8.79%)	118,334 (10.53%)	
	Burn, n (%)	17,977 (1.75%)	17,886 (1.65%)	18698 (1.66%)	
	Other, n (%)	12,990 (1.27%)	12,727 (1.17%)	13,517 (1.20%)	
Mechanism of injury	Cut/pierce, n (%)	4,639 (0.44%)	5,188 (0.47%)	6,104 (0.54%)	<0.0001
	Fall: Ground level, n (%)	152,627 (14.62%)	165,474 (15.08%)	172,111 (15.16%)	
	Fall: Height, n (%)	515 (0.05%)	585 (0.05%)	592 (0.05%)	
	Burn, n (%)	291 (0.03%)	265 (0.02%)	257 (0.02%)	
	Gunshot wound, n (%)	1,143 (0.11%)	1,570 (0.14%)	2210 (0.19%)	
	Motor vehicle accident (MVA): Occupant, n (%)	158,254 (15.16%)	158,452 (14.44%)	154,614 (13.62%)	
	MVA: Motorcycle, n (%)	2,312 (0.22%)	2,371 (0.22%)	2,639 (0.23%)	
	MVA: Pedestrian, n (%)	659 (0.06%)	587 (0.05%)	500 (0.04%)	
	MVA: Other, n (%)	3,432 (0.33%)	3,223 (0.29%)	3,383 (0.30%)	
	Assault, n (%)	26,044 (2.50%)	26,369 (2.40%)	25,563 (2.25%)	
	Other mechanisms, n (%)	693,820 (66.47%)	733,106 (66.82%)	767,045 (67.58%)	

Injury type

Injury type distributions (blunt, penetrating, burn) differed significantly across years. Penetrating injuries increased to 10.53% (118,334/2020) from 8.79% (95,484/2019) and 8.92% (91,365/2018). Blunt injuries decreased to 86.60% (973,047/2020) from 88.30% (960,230/2019) and 88.00% (902,488/2018). Burn injuries were 1.66% (18,698/2020), compared with 1.65% (17,886/2019) and 1.75% (17,977/2018) (Table 1).

Mechanism of injury 

Mechanisms of injury differed significantly across years. Cutting/piercing injuries comprised 0.54% (6,104/2020) versus 0.47% (5,188/2019) and 0.44% (4,639/2018). Ground-level falls were 15.16% (172,111/2020) versus 15.08% (165,474/2019) and 14.62% (152,627/2018). Gunshot wounds were 0.19% (2,210/2020) versus 0.14% (1,570/2019) and 0.11% (1,143/2018). Motor vehicle accident (MVA) occupants accounted for 13.62% (154,614/2020) versus 14.44% (158,452/2019) and 15.10% (158,254/2018). Assaults comprised 2.25% (25,563/2020) versus 2.40% (26,369/2019) and 2.50% (26,044/2018) as shown in Table 1.

Presentation and early care 

Vital signs at presentation differed significantly between pre-COVID-19 and COVID-19 years. A Glasgow coma scale (GCS) score <9 occurred in 49.32% (559,795/2020), compared with 49.93% (547,835/2019) and 48.87% (510,120/2018). Blood transfusion within four hours differed significantly across years: 4.33% (44,273/2020), 3.95% (38,866/2019), and 3.89% (36,431/2018). Injury severity score (ISS) <15 was observed in 84.28% (956,647/2020) versus 84.87% (931,185/2019) and 84.50% (881,960/2018) (Table 2).

**Table 2:** Presentation/early care and comorbidities by year The p-values were calculated using chi-square tests. GCS: Glasgow coma scale; ISS: Injury severity score; COPD: Chronic obstructive pulmonary disease

Variable	Year			p-value
	2018	2019	2020	
Oxygen saturation <94%, n (%)	621,044 (59.50%)	654,836 (59.68%)	659,313 (58.09%)	<0.0001
Blood pressure <90 mmHg, n (%)	502,843 (48.18%)	536,759 (48.92%)	552,071 (48.64%)	<0.0001
GCS <9, n (%)	510,120 (48.87%)	547,835 (49.93%)	559,795 (49.32%)	<0.0001
Blood transfusion within four hours, n (%)	36,431 (3.89%)	38,866 (3.95%)	44,273 (4.33%)	<0.0001
ISS <15, n (%)	881,960 (84.50%)	931,185 (84.87%)	956,647 (84.28%)	<0.0001
Hypertension, n (%)	350,241 (33.56%)	378,621 (34.51%)	389,615 (34.33%)	<0.0001
Alcohol use disorder, n (%)	49,580 (4.75%)	46,196 (4.21%)	63,495 (5.59%)	<0.0001
Angina, n (%)	1,514 (0.15%)	2,039 (0.19%)	2,179 (0.19%)	<0.0001
Anticoagulant use, n (%)	101,036 (9.68%)	119,941 (10.93%)	126,213 (11.12%)	<0.0001
Bleeding disorder, n (%)	10,826 (1.04%)	10,574 (0.96%)	10,206 (0.90%)	<0.0001
Currently on chemotherapy, n (%)	4,281 (0.41%)	5,017 (0.46%)	5,197 (0.46%)	<0.0001
Cirrhosis, n (%)	8,909 (0.85%)	9,994 (0.91%)	11,320 (1.00%)	<0.0001
Chronic obstructive pulmonary disease (COPD), n (%)	68,944 (6.61%)	73,522 (6.70%)	76,887 (6.77%)	<0.0001
Cerebrovascular accident, n (%)	27,990 (2.68%)	29,587 (2.70%)	29,556 (2.60%)	<0.0001
Dementia, n (%)	58,908 (5.64%)	65,249 (5.95%)	7,0391 (6.20%)	<0.0001
Diabetes, n (%)	137,580 (13.18%)	147,328 (13.43%)	151,685 (13.36%)	<0.0001
Disseminated cancer, n (%)	6,378 (0.61%)	6,066 (0.55%)	6,458 (0.57%)	<0.0001
Functionally dependent, n (%)	86,435 (8.28%)	108,181 (9.86%)	121,771 (10.73%)	<0.0001
Congestive heart failure, n (%)	43,830 (4.20%)	48,441 (4.42%)	52,674 (4.64%)	<0.0001
Myocardial infarction history, n (%)	8,600 (0.82%)	7,567 (0.69%)	7,380 (0.65%)	<0.0001
Peripheral artery disease, n (%)	7,506 (0.72%)	9,458 (0.86%)	11,344 (1.00%)	<0.0001
Mental health disorder, n (%)	103,224 (9.89%)	106,360 (9.69%)	119,231 (10.50%)	<0.0001
Renal failure, n (%)	17,084 (1.64%)	17,723 (1.62%)	19,346 (1.70%)	<0.0001
Smoker, n (%)	190,723 (18.27%)	198,851 (18.12%)	214,876 (18.93%)	<0.0001
Steroid use, n (%)	10,182 (0.98%)	11,664 (1.06%)	11,561 (1.02%)	<0.0001
Substance use disorder, n (%)	68,614 (6.57%)	47,390 (4.32%)	67,664 (5.96%)	<0.0001

Comorbidities and patient characteristics 

Comorbidities and patient characteristics differed significantly between pre-COVID-19 and COVID-19 years. Alcohol use disorder was present in 5.59% (63,495/2020) versus 4.21% (46,196/2019) and 4.75% (49,580/2018). Mental health disorders were 10.50% (119,231/2020) versus 9.69% (106,360/2019) and 9.89% (103,224/2018). Substance use disorders were 5.96% (67,664/2020), versus 4.32% (47,390/2019) and 6.57% (68,614/2018). A history of smoking was reported in 18.93% (214,876/2020,) versus 18.12% (198,851/2019) and 18.27% (190,723/2018). Functional dependence was 10.73% (121,771/2020) versus 9.86% (108,181/2019) and 8.28% (86,435/2018) as shown in Table 2.

In-hospital events

Rates of deep surgical site infection (SSI), deep vein thrombosis (DVT), compartment syndrome, organ-space SSI, osteomyelitis, and stroke did not differ significantly across 2018-2020. Alcohol withdrawal differed significantly: 0.63% (7,099/2020) versus 0.58% (6,320/2019) and 0.58% (6,076/2018). Cardiac arrest also differed significantly, with rates of 0.71% (8,019/2020), 0.63% (6,963/2019), and 0.63% (6,573/2018). Pulmonary embolism (PE) differed significantly: 0.25% (2,788/2020) versus 0.22% (2,410/2019) and 0.25% (2,600/2018) (Table 3). Unplanned intubations differed significantly across years: 0.84% (9,501/2020) versus 0.82% (9,014/2019) and 0.81% (8,439/2018). Unplanned ICU admissions also differed significantly: 1.42% (16,083/2020) versus 1.41% (15,450/2019) and 1.32% (13,747/2018).

**Table 3:** In-hospital events by year The p-values were calculated using chi-square tests. CAUTI: Catheter-associated urinary tract infection; CLABSI: Central-line–associated bloodstream infection; SSI: Surgical site infection; DVT: Deep vein thrombosis; PE: Pulmonary embolism; AKI: Acute kidney injury; MI: Myocardial infarction; ARDS: Acute respiratory distress syndrome; OR: Operating room; VAP: Ventilator-associated pneumonia

Event	Year			p-value
	2018	2019	2020	
Alcohol withdrawal, n (%)	6,076 (0.58%)	6,320 (0.58%)	7,099 (0.63%)	<0.0001
Cardiac arrest, n (%)	6,573 (0.63%)	6,963 (0.63%)	8,019 (0.71%)	<0.0001
Catheter-associated urinary tract infection (CAUTI), n (%)	1,759 (0.17%)	1,441 (0.13%)	1,281 (0.11%)	<0.0001
Central-line–associated bloodstream infection (CLABSI), n (%)	304 (0.03%)	237 (0.02%)	245 (0.02%)	0.0002
Deep SSI, n (%)	852 (0.08%)	808 (0.07%)	856 (0.08%)	0.0871
DVT, n (%)	4,871 (0.47%)	4,902 (0.45%)	5,232 (0.46%)	0.0830
PE, n (%)	2,600 (0.25%)	2,410 (0.22%)	2,788 (0.25%)	<0.0001
Compartment syndrome, n (%)	771 (0.07%)	828 (0.08%)	877 (0.08%)	0.6589
Unplanned intubation, n (%)	8,439 (0.81%)	9,014 (0.82%)	9,501 (0.84%)	0.0653
Acute kidney injury (AKI), n (%)	4,279 (0.41%)	4,307 (0.39%)	4,902 (0.43%)	<0.0001
Myocardial infarction (MI) (in-hospital), n (%)	1,460 (0.14%)	1,543 (0.14%)	1,280 (0.11%)	<0.0001
Organ-space SSI, n (%)	512 (0.05%)	540 (0.05%)	572 (0.05%)	0.8870
Osteomyelitis, n (%)	172 (0.02%)	155 (0.01%)	185 (0.02%)	0.3022
ARDS (acute respiratory distress syndrome), n (%)	2,388 (0.23%)	2,078 (0.19%)	1,975 (0.17%)	<0.0001
Return to operating room (OR), n (%)	4,035 (0.39%)	4,128 (0.38%)	0 (0.00%)	<0.0001
Sepsis, n (%)	2,458 (0.24%)	2,102 (0.19%)	2,477 (0.22%)	<0.0001
Stroke, n (%)	2,235 (0.21%)	2,264 (0.21%)	2,488 (0.22%)	0.1116
Superficial SSI, n (%)	775 (0.07%)	733 (0.07%)	752 (0.07%)	0.0454
Pressure ulcer, n (%)	3,226 (0.31%)	3,564 (0.32%)	3,858 (0.34%)	0.0003
Unplanned ICU admission, n (%)	13,747 (1.32%)	15,450 (1.41%)	16,083 (1.42%)	<0.0001
Ventilator-associated pneumonia (VAP), n (%)	3,580 (0.34%)	3,131 (0.29%)	3,248 (0.29%)	<0.0001

Procedures and outcomes

The proportions of thoracotomy and laparotomy did not differ significantly across years. Mean hospital length of stay did not differ significantly, but there was a statistically significant difference in ICU length of stay. Mortality differed significantly: 3.07% (29,223/2020) versus 2.89% (26,689/2019) and 3.00% (26,513/2018) (Table 4).

**Table 4:** Disposition, procedures, and length of stay by year The p-values were calculated using chi-square tests for categorical variables (disposition and procedures) and the Kruskal–Wallis test for lengths of stay. The p-value shown pertains to the overall comparison across subcategories. LOS: Length of stay

Variable		Year			p-value
		2018	2019	2020	
Disposition	Death, n (%)	26,513 (3.00%)	26,689 (2.89%)	29,223 (3.07%)	<0.0001
	Transferred out, n (%)	19,593 (2.22%)	19,785 (2.14%)	18,841 (1.98%)	
	Discharged to rehab, n (%)	95,665 (10.83%)	99,503 (10.79%)	98,396 (10.35%)	
Procedures	Thoracotomy, n (%)	2,445 (6.75%)	2,606 (6.76%)	3,225 (6.88%)	0.1459
	Laparotomy, n (%)	9,356 (25.83%)	9,669 (25.07%)	11,845 (25.26%)	
Length of stay, days (median (IQR))	Hospital LOS	3 (2-6)	3 (2-6)	3 (2-6)	0.0871
	ICU LOS	3 (2-5)	3 (2-5)	3 (2-5)	<0.0001

## Discussion

Our findings show a higher proportion of Black patients in 2020, consistent with prior reports, particularly from medically underserved regions of the US [3-6]. These data raise concerns about racial disparities in the broader effects of the pandemic on health and well-being. Strassle et al. reported that Black/African American individuals faced higher risks of assault and hospitalization during the COVID-19 era [7].

We also observed an increased proportion of penetrating trauma (e.g., cutting, piercing, and gunshot wounds) in 2020, aligning with multiple studies that documented similar shifts during the pandemic [3,4,8-15]. 'Stay-at-home' orders and pandemic-related stress may have contributed to this trend. In contrast, blunt trauma decreased slightly in 2020. Although Mokhtari et al. suggested that local socioeconomic and cultural factors may underlie the rising incidence of penetrating trauma early in the pandemic [16], the increase has been observed across many published analyses [3,4,8-15]. Notably, our data did not show an increase in assaults during the pandemic year.

Early care indicators also shifted. A greater proportion of patients received blood within four hours of arrival in 2020, suggesting that those who presented were more severely injured. This interpretation is consistent with the lower proportion of patients with ISS <15 in 2020 compared with 2019 and 2018.

We observed higher proportions of alcohol use disorder, mental health disorders, and smoking history in 2020 than in prior years, consistent with other reports [13,17]. Alcohol sales rose during the pandemic, and social unrest and prolonged stress may have exacerbated alcohol misuse and mental health conditions. Zhai et al. similarly reported increased diagnoses of alcohol use and psychiatric disorders among trauma patients during the COVID-19 period [18].

In-hospital events also shifted: cardiac arrest and PE were more frequent in 2020 than in prior years, consistent with a generally sicker inpatient population. Whether severe acute respiratory syndrome coronavirus 2 (SARS-CoV-2) infection directly contributed to these events cannot be determined from our data; however, it remains a plausible possibility. Unplanned ICU admissions and unplanned intubations also increased in 2020. Several studies have associated COVID-19 infection with higher mortality and specific morbidities [19-21]. In contrast, the frequencies of laparotomy and thoracotomy were stable, suggesting that operative management for trauma and intra-abdominal emergencies was not markedly altered. Overall mortality was higher in 2020, consistent with other analyses from the pandemic period [22].

In sum, penetrating trauma increased during the pandemic year, and several markers indicated sicker patients and more severe complications. Together with prior literature, these findings underscore how a pandemic can strain trauma systems in the US and globally and highlight the importance of resilient systems to maintain trauma care during public health emergencies.

Limitations 

This retrospective analysis of the TQIP has several limitations. First, although we analyzed a large, multicenter dataset, participation in TQIP is voluntary and may not represent all US trauma centers or regions, introducing selection bias and limiting generalizability. Second, the secondary use of registry data is vulnerable to coding variability, misclassification (e.g., mechanism, comorbidities, complications), and missing or incomplete fields, despite the use of NTDS definitions. Third, the dataset does not capture key COVID-19-specific variables such as SARS-CoV-2 test results, vaccination status, local incidence, or hospital strain (e.g., staffing shortages, bed availability) that could confound associations between the pandemic period and observed outcomes. Fourth, the analysis focuses on descriptive comparisons across years and cannot establish causality; unmeasured confounders (e.g., socioeconomic shifts, changes in healthcare-seeking behavior, law-enforcement activity, and regional policy timing and intensity) may account for part of the observed differences. Fifth, within-year temporal granularity is limited; we could not distinguish between the early and late phases of 2020 or evaluate seasonal patterns. Sixth, we did not examine out-of-hospital deaths or prehospital triage changes, which could alter the case mix that reaches hospitals. Seventh, institutional practice changes during the pandemic (e.g., thresholds for ICU admission, blood product utilization, or operative decision-making) were not measured and may vary across centers. Eighth, multiple year-to-year comparisons increase the risk of a type I error, and a large sample may yield statistically significant but clinically modest differences. Finally, although the 2021 data were available, primary comparisons emphasized pre-pandemic versus early pandemic years; therefore, the findings may not accurately reflect later pandemic phases or post-vaccine periods.

## Conclusions

This study used the TQIP to examine national trends in trauma care before and during the COVID-19 pandemic, analyzing whether patient demographics, injury mechanisms, injury severity, comorbidities, in-hospital events, and outcomes differed across these periods. We observed clear shifts during the pandemic year: penetrating mechanisms became more common, presenting patients appeared more severely injured, unplanned critical-care interventions increased, and mortality rates were higher. Patterns in patient demographics also indicated disproportionate impacts on Black/African American patients, highlighting concerns about exacerbated health disparities. These findings identify the areas where trauma systems are most vulnerable during public health emergencies and highlight actionable priorities. Health systems can use this evidence to enhance surge preparedness, ensure adequate staffing, blood products, and critical care capacity, integrate violence prevention, mental health, and substance use screening into trauma pathways, and design equity-focused policies that safeguard access and outcomes for underserved populations. By clarifying how trauma epidemiology and care needs to shift under pandemic conditions, this study provides a practical foundation for readiness planning and more equitable, resilient trauma care.
